# Gypenosides Attenuate Lipopolysaccharide-Induced Neuroinflammation and Memory Impairment in Rats

**DOI:** 10.1155/2018/4183670

**Published:** 2018-06-19

**Authors:** Bombi Lee, Insop Shim, Hyejung Lee

**Affiliations:** ^1^Acupuncture and Meridian Science Research Center, College of Korean Medicine, Kyung Hee University, Seoul 02447, Republic of Korea; ^2^Department of Physiology, College of Medicine, Kyung Hee University, Seoul 02447, Republic of Korea

## Abstract

Neuroinflammation is deliberated a major factor in various neurodegenerative diseases. Gypenosides (GPS) have pharmacological properties with multiple beneficial effects including anti-inflammatory, antioxidative, and protective properties. In the present study, whether GPS could improve cognitive dysfunction and chronic inflammation caused by injecting lipopolysaccharide (LPS) in the hippocampus was investigated. Effects of GPS on inflammatory factors in the hippocampus and the downstream mechanisms of these effects were also examined. Induction of LPS into the lateral ventricle caused inflammatory reactions and memory impairment on the rats. Every day treatment of GPS (25, 50, and 100 mg/kg) for 21 consecutive days attenuated spatial recognition, discrimination, and memory deficits. GPS treatment significantly decreased proinflammatory mediators such as interleukin-6 (IL-6), interleukin-1*β* (IL-1*β*), and nuclear factor-kappaB (NF-*κ*B) levels in the brain. Furthermore, GPS reduced LPS-induced elevated levels of inducible nitric oxide synthase (iNOS) and toll-like receptor 4 (TLR4) mRNA and inhibition of brain-derived neurotrophic factor (BDNF) mRNA level. Collectively, these results showed that GPS may improve cognitive function and provide a potential therapy for memory impairment caused by neuroinflammation. Based on these, GPS may be effective in inhibiting the progress of neurodegenerative diseases by improving memory functions due to its anti-inflammatory activities and appropriate modulation of NF-*κ*B/iNOS/TLR4/BDNF.

## 1. Introduction

Neuroinflammation, which includes inflammation of the central nervous system (CNS), has been considered a frequent cause of different neurological disorders including ischemic stroke, Parkinson's disease (PD), and Alzheimer disease (AD) [1]. In addition, the immune system undergoes several important changes during the aging process, which renders older people more vulnerable to infections [2, 3]. Additionally, sepsis after severe infection is followed by lasting cognitive dysfunction, particularly in older individuals [4]. More recently, considerable evidence has been shown that neuroinflammation plays an important role in the increase of various neurocognitive diseases, such as postsepsis encephalopathy [5]. Therefore, many studies have demonstrated that neuroinflammatory and sustained increases in various proinflammatory mediators including interleukin-6 (IL-6), interleukin-1*β* (IL-1*β*), and tumor necrosis factor-*α* (TNF-*α*) in the CNS are closely related to the memory impairment primarily related to progress of AD pathogenesis [6, 7]. Lipopolysaccharide (LPS) is a noncatching element of the external membranes of Gram-negative bacteria that regulates proinflammatory cytokines [8]. Systemic injection of LPS is associated with neuroinflammation in the hippocampus critical for learning and memory processes [9], leading to cognitive dysfunction [10]. Therefore, LPS has been often used to research the biochemical mechanisms of cognitive dysfunction due to inflammation and to develop targeted remedial for neurological symptoms in animal model [11]. LPS stimulates proinflammatory cytokine cascades by toll-like receptor 4 (TLR4) causing production of many proinflammatory cytokines including IL-6, IL-1*β*, and TNF-*α*, and activation of the nuclear factor-kappaB (NF-*κ*B) system [12, 13]. Currently, nutraceuticals targeting neuroinflammatory mediators have been suggested as novel therapeutic tools for the management of neurodegenerative and neuroinflammatory disorders [14, 15].

Some studies have shown that long-term use of nonsteroidal anti-inflammatory drugs (NSAIDs) inhibits cognitive diminution in elderly individuals identified with AD [16]. But, repetitive treatment using NSAIDs can cause kidney toxicity, occasional liver, and gastrointestinal side effects [17]. These side effects have prompted the advance of new NSAIDs that are safer for long-term treatment [18]. The use of herbal medicines or natural products for treating Alzheimer type-dementia and related disorders exhibiting cognitive memory impairment and neuroinflammation has been suggested in recent studies [19].

Gypenosides (GPS), the saponin extract derived from* Gynostemma pentaphyllum*, is a well-known herbal medicinal plant in Southeast Asia used for the treatment of hyperlipidemia, cardiovascular disease, and chronic inflammation [20]. The active component of GPS is the hydroxyl-group attached to the twentieth or twenty-first carbon in the dammarane-type ring [21]. Using modern scientific methods, GPS was shown to have a diversity of pharmacological properties, including neuroprotective, cardioprotective, antiaging, and antitumor effects [22–25]. In several studies, GPS exerted antioxidative capacities in primary cultures of cortical cells in glutamate-treated rat, and in the hippocampal CA1 region and cortex in a chronic cerebral hypoperfusion rat model [21, 26]. In addition, GPS was shown to improve superoxide dismutase (SOD) motion in tissues and serum, increase antioxidant abilities, and decrease levels of oxidized low-consistency lipoprotein [27]. In the rat heart, GPS alleviated myocardial ischemia-reperfusion injury by preserving mitochondrial function and attenuating oxidative stress [28]. In several studies, GPS was shown to exert neuroprotective effects on MPP^+^-induced dopaminergic neurons in primary nigral cultures [29, 30]. Reportedly, GPS improved the affective symptoms in a 1-methyl-4-phenyl-1,2,3,6-tetrahydrophyfidine (MPTP)-induced PD in mouse model [31]. Moreover, GPS exerted effects opposed to levodopa (L-DOPA)-induced dyskinesia in a 6-hydroxydopamine (6-OHDA)-induced PD in rat model [31, 32]. These findings imply that GPS could serve as a promising therapeutic option for sepsis-induced neuroinflammation and related cognitive decline, especially in elderly populations, and remains minimally investigated.

In this research, anti-inflammatory efficacies of GPS on memory and learning functions in LPS-treated neuroinflammation rats as measured by execution on the Morris water maze (MWM) test and the object recognition task (ORT) were investigated. Furthermore, we also investigated how these effects were associated with the molecular modulation of neuroinflammation in the neural mechanisms underlying the improvement of GPS in the memory impairment.

## 2. Methods

### 2.1. Animals

Male seven-week-old SD rats (weights: 200-220 g, Samtako Animal Co., Seoul, Korea) were used in this study. The vivarium room was kept on a 12-h light/dark cycle (lights on at 9:00, lights off at 21:00) under relative humidity of 55 ± 10 % and a controlled temperature at 22 ± 5°C. All rats were caged for 7 days to acclimatize before beginning the experimental protocol. All methods and procedures were approved by the Animal Care and Use Committee of Kyung Hee University [KHUASP(SE)-15-115]. Experimental procedures were performed according to the Guide for the Care and Use of Laboratory Animals.

### 2.2. LPS Treatment

Gypenosides, ibuprofen (IBU), and LPS (Escherichia coli; O127:B8) were obtained by Sigma-Aldrich Company (Sigma-Aldrich Co., St. Louis, MO, USA). LPS administrated intracerebroventricularly (i.c.v.) into the lateral ventricle of the rat brain according to the method of Guo et al. [33] controlled anesthesia with 50 mg/kg sodium pentobarbital intraperitoneal injection (i.p.). Injection of LPS was delivered at a rate of 2 *μ*L/1 min (total 5 min) and injection needles were left in place an additional 5 min. The sham control animals were administrated the vehicle (i.c.v. and i.p.) instead of one of the drug solutions.

### 2.3. Experimental Groups

**Figure 1:**
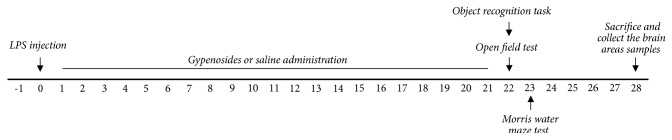
Experimental schedule of lesion generation, GPS administration, and behavioral tests in rats. GPS; gypenosides, LPS; lipopolysaccharide, OFT; open field test, ORT; object recognition task, and MWM; Morris water maze test.

Rats were casually separated in 6 groups of 6-7 individuals as follows: SAL-injected control group (SAL group, n = 6), LPS-injected plus saline-treated group (LPS group as a negative control, n = 6), LPS-injected plus 25 mg/kg GPS-treated group (LPS + GPS25 group, n = 6), LPS-injected plus 50 mg/kg GPS-treated group (LPS + GPS50 group, n = 6), LPS-injected plus 100 mg/kg GPS-treated group (LPS + GPS100 group, n = 7), and LPS-injected plus 40 mg/kg IBU-treated (LPS + IBU group as a positive control, n = 6). The whole schedule of behavioral examinations and drug administration are shown in Figure 1.

### 2.4. Object Recognition Task (ORT)

The novel ORT was used to assess the cognitive capability of animals. Briefly, the apparatus consists of square box made of black painted wood having dimensions of 45 × 45 × 45 cm^3^. The objects to be discriminated were two similar wooden block toys as familiar objects (A1 and A2) in order to make them heavy enough so that rats could not be able to move them, and different shape and different color of wooden block toys as a novel object (B). On day one in order to be habituated, the rats were adapted to the object recognition box for 10 min. Next day 24 h after habituation, rats were positioned inside the box with two familiar objects (A1 and A2) and were allowed to explore the objects for 5 min. During the test phase, rats housed to the testing chamber, where it was exposed to one novel object (B) and one of the familiar objects for 5 min. The sniffing time for the novel and familiar object was measured. The discrimination index is measured of discrimination between the familiar and the novel object accurate for exploration. It is calculated as (time spent on novel object – time spent on familiar object)/(time spent on novel object + time spent on familiar object) [34].

### 2.5. Morris Water Maze Test (MWM)

Spatial learning and memory of rats were evaluated in MWM test as published previously [35]. MWM testing included a spatial probe test and a place navigation test. The MWM consisted of a circular pool (200 cm diameter and 50 cm deep) filled with water 22 ± 2°C and an escape platform (10 cm diameter) submerged 1 cm below the surface of the water. Swimming activity of the rats was monitored via a video camera mounted overhead and automatically recorded via a video tacking system. The rats underwent three training trials per day for 5 consecutive days. Each trial was terminated when the rat found the platform or after 180 s. The shorter escape latency, the stronger spatial learning ability. On the 6th day, the platform was removed. In this probe trial, the each trial was 60 s in duration. The swimming path length, swimming speed, and time spent in the target quadrant were measured.

### 2.6. Open Field Test (OFT)

The OFT was based on previous study [36]. In the dimly lit room, each rat was housed individually in a square black plexiglass apparatus (60 × 60 × 30 cm) and tracked by a video tracking system for 5 min. Locomotion was analyzed by the distance and speed of movements and observed by a computerized video tracking analysis program S-MART (PanLab Co., Barcelona, Spain). The number of rearing was also manually scored by examining the records in the OFT.

### 2.7. COX-2, TNF-*α*, IL-1*β*, IL-6, and NF-*κ*B Measurement

Twenty-eight day after inducing LPS, cyclooxygenase (COX)-2, TNF-*α*, IL-1*β*, IL-6, and NF-*κ*B concentrations were assayed in the hippocampus using a method described previously [36]. Three rats from each group were anesthetized with 1.2 % isoflurane, and the brains were removed. The COX-2, TNF-*α*, IL-1*β*, IL-6, and NF-*κ*B concentrations were assessed by an enzyme-linked immunoassay (ELISA) according to the manufacturer's method. COX-2, TNF-*α*, IL-1*β*, IL-6, and NF-*κ*B were purchased from Abcam (Cambridge, MA, USA).

### 2.8. Total RNA Isolation and RT-PCR Analysis

The expression levels of inducible brain-derived neurotrophic factor (BDNF), TLR4, and nitric oxide synthase (iNOS) mRNA were evaluated by reverse transcription polymerase chain reaction (RT-PCR) according to previous study [36]. Total RNA was extracted from the hippocampus, using Trizol reagent, and cDNA was synthesized with reverse transcriptase (Takara, Kyoto, Japan). Data were normalized against glyceraldehyde 3-phosphate dehydrogenase (GAPDH) expression in the corresponding sample.

### 2.9. Statistical Analysis

All data are expressed as mean ± SEM. The data were analyzed with SPSS 13.0 (Chicago, IL, USA). Data were analyzed by the multiple way of analysis of variance (ANOVA) and Tukey's* post hoc* tests. Statistical significance at* p value* < 0.05 has been given symbols in each figures.

## 3. Results

### 3.1. Effect of GPS on LPS-Induced Body Weight Loss

**Figure 2:**
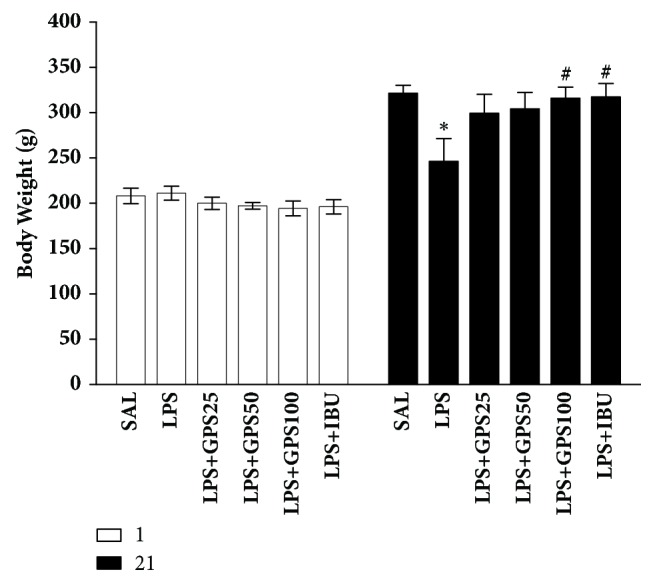
Effects of GPS on body weight gain on the first day prior to LPS injection and on day 21 after LPS injection. ^*∗*^*p* < 0.05 versus SAL group; ^#^*p* < 0.05 versus LPS group.

After injection of LPS, body weight gain was measured for 21 days (Figure 2). Rats exposed to LPS began to lose body weight on day 1, and this LPS-induced reduction of body weight is sustained for 21 days for a while without restoring to normal level, or even exacerbated in some cases [37]. Compared with the saline-treated (SAL) group, the rats in the LPS group induced body weight loss for 21 days (*p* < 0.05). The body weight in the LPS + GPS100 group showed a significant inhibition of the reduction in body weight gain compared with that in the LPS group for 21 days (*p* < 0.05), indicating that recovery of body weight in the LPS + GPS100 group was closely associated with those in the LPS + IBU group.

### 3.2. Effects of GPS on LPS-Induced Memory Impairment

**Figure 3:**
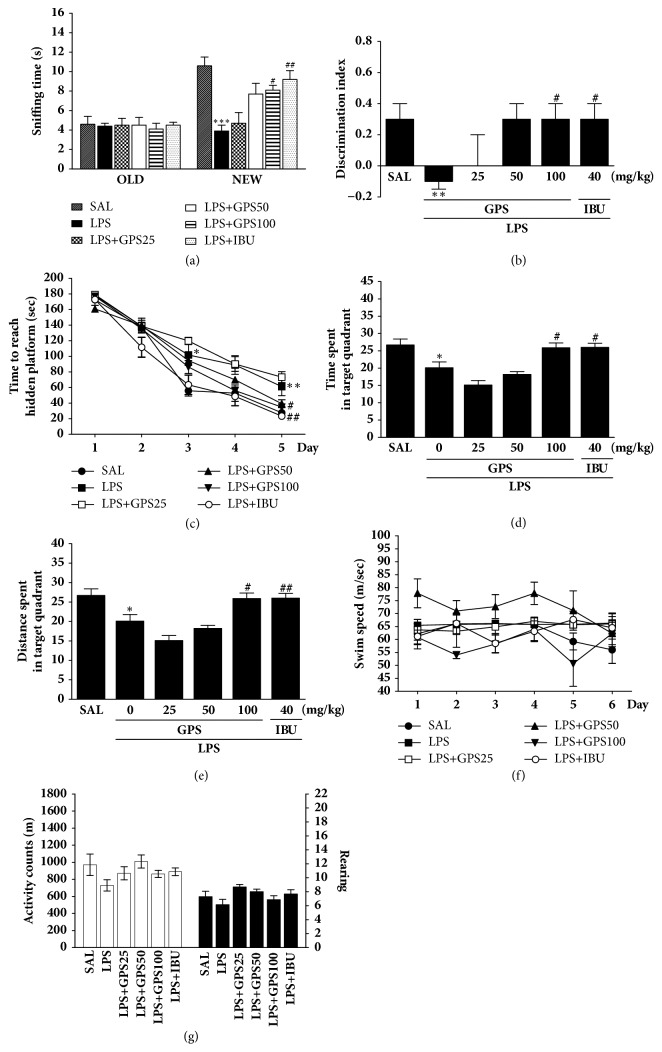
Effects of GPS on recognition memory were evaluated using a novel ORT in which we analyzed the times taken to sniff familiar and novel objects during a 5-min choice trial (A) and the ability to discriminate between familiar and novel objects (B). The MWM test was used to evaluate the effect of GPS on spatial learning and memory; we analyzed the time taken to escape from water (latency) during acquisition trials using a submerged platform (C), the percentages of time spent in the target quadrant (D), the proportion of the total distance traversed in the target quadrant (E), and swimming speed (F). The OFT was used to evaluate the effect of GPS on locomotor activity and total number of rearings (G). ^*∗*^*p* < 0.05, ^*∗∗*^*p* < 0.01, ^*∗∗∗*^*p* < 0.001 versus SAL group; ^#^*p* < 0.05, ^##^*p* < 0.05 versus LPS group.

Recognition memory was indicated in terms of the sniffing times of familiar and novel objects and by calculation of discrimination indices (Figure 3). No significant differences in the sniffing times on the training day were detected between experimental groups, by two-way ANOVA [F(5,36) = 0.555, p = 0.733]. During the retention trial, there was a significant difference in novel object exploration [F(5,36) = 11.733, p < 0.001]. These sniffing times were significantly reduced following LPS injection, compared to control (*p* < 0.001). The PTSD + GPS100 group exhibited increased novel object sniffing time compared to the LPS group (*p* < 0.05).* Post hoc* examination with Tukey's test revealed that the discrimination index of LPS group was significantly lower than that of SAL group (*p* < 0.01). In addition, the discrimination index of LPS group that received GPS (100 mg/kg) was significantly higher than that of rats in the LPS-only group (*p* < 0.05). The LPS + GPS100 group in recovery of recognition was similar to those in the LPS + IBU group. In MWM test, LPS-induced rats were weak to learn during acquisition trial and retention trial. However, the learning and memory abilities of LPS-injected rats were significantly impaired compared with the SAL group (*p* < 0.05 on day 3 and *p* < 0.01 on day 5). A significant decrease in escape latency was observed in the LPS + GPS100 group compared to the LPS group (*p* < 0.05). In the probe trial of the MWM test, injection of LPS had a significant effect on the platform-cross number compared with the SAL group (*p* < 0.05). Compared with LPS group, GPS-treated rats displayed more platform-cross number (*p* < 0.05). The swimming latency in the LPS + GPS100 group was similar to that in the LPS + IBU group. The LPS group did not differ significantly from the other groups in the mean swimming speed.

The LPS-treated rats demonstrated considerable motor function as measured by the locomotion and exploration as total number of rearing behaviors in the OFT. We found no significant difference among LPS-treated, saline-treated, and GPS-treated rats in terms of locomotor activity [F(5,36) = 1.975, *p* = 0.110] or the total number of rearing behaviors [F(5,36) = 0.707, *p* = 0.622].

### 3.3. Effects of GPS on LPS-Induced Changes in Inflammatory Mediators in the Hippocampus

**Figure 4:**
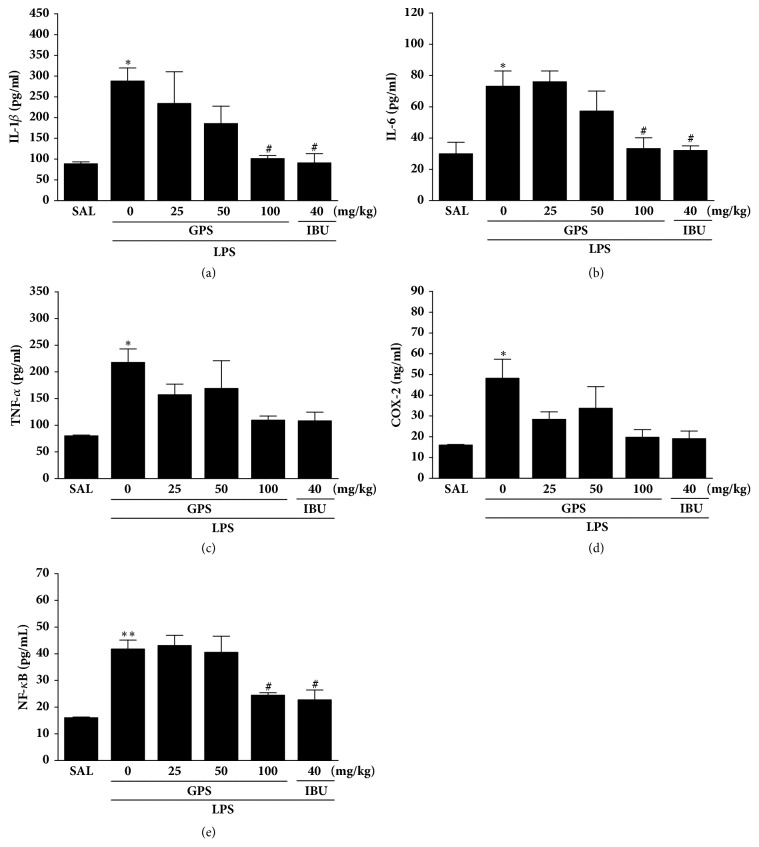
Effects of GPS on interleukin-1*β* (IL-1*β*), interleukin-6 (IL-6), tumor necrosis factor-*α* (TNF-*α*), cyclooxygenase-2 (COX-2), and nuclear factor-kappaB (NF-*κ*B) concentrations in the brain of rats exposed to LPS for 28 consecutive days. ^*∗*^*p* < 0.05, ^*∗∗*^*p* < 0.01 versus SAL group; ^#^*p* < 0.05 versus LPS group.

Following the behavioral test, brain tissues were analyzed to investigate the effect of administration of GPS on the levels of the proinflammatory markers activated by LPS-induced inflammation in the hippocampus (Figure 4). It shows that the hippocampus levels of COX-2, TNF-*α*, IL-1*β*, IL-6, and NF-*κ*B were significantly different when the group were compared. These results showed a significant increase in the levels of IL-1*β* in the hippocampus of the LPS groups compared to those in the SAL group (*p* < 0.05). Administration of GPS significantly decreased the LPS-induced increase in IL-1*β* concentration in the hippocampus compared to those in the LPS group (*p* < 0.05). After GPS administration, the levels of IL-6 in the hippocampus decreased significantly to 45.49 % of those in the LPS group (*p* < 0.05). It also indicated that the recovery of the IL-1*β* and IL-6 concentrations in the hippocampus in the LPS + IBU group almost was similar to that with the LPS + GPS100 group.

It showed that LPS injection significantly increased the TNF-*α* and COX-2 concentrations in the hippocampus of rats by 272.02 % and 301.19 % compared to the SAL group (*p* < 0.05), respectively. However, administration of GPS inhibited the LPS-induced increase in the TNF-*α* and COX-2 expressions in the hippocampus, respectively, although this result was only marginally statistically.

Also, these results showed a significant increase in the levels of NF-*κ*B in the hippocampus of the LPS groups compared to the SAL group (*p* < 0.01). Administration of GPS significantly decreased the LPS-induced increase in NF-*κ*B levels in the hippocampus compared to the LPS group (*p* < 0.05).

### 3.4. Effects of GPS on LPS-Induced Expression of iNOS, TLR4, and BDNF mRNAs in the Hippocampus

**Figure 5:**
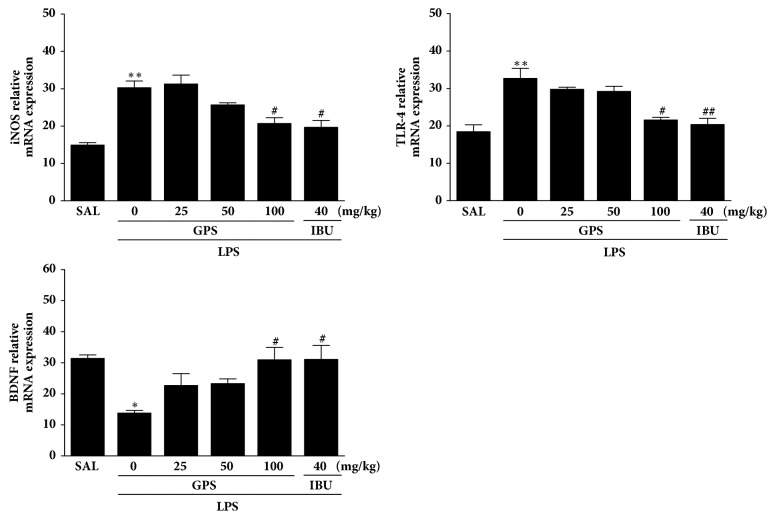
Effects of GPS on the expression of inducible nitric oxide synthase (iNOS), toll-like receptor 4 (TLR4), and brain-derived neurotrophic factor (BDNF) mRNAs in rats with LPS-induced hippocampal impairment. The expression levels of iNOS, TLR4, and BDNF mRNAs were normalized to glyceraldehyde 3-phosphate dehydrogenase (GAPDH) mRNA as an internal control. ^*∗*^*p* < 0.05 and ^*∗∗*^*p* < 0.01 versus SAL group; ^#^*p* < 0.05, ^##^*p* < 0.01 versus LPS group.

iNOS, TLR4, and BDNF mRNA levels were assessed to inspect the effect of GPS on the expression of iNOS, TLR4, and BDNF mRNA in the hippocampus (Figure 5). The mRNA of iNOS in the LPS group increased significantly compared to the SAL group (*p* < 0.01). The increased expression of iNOS mRNA in the LPS group was significantly decreased by treatment of 100 mg/kg of GPS (*p* < 0.05). TLR4 mRNA expression in the LPS group was increased significantly compared to the SAL group (*p* < 0.01). The increased expression of TLR4 mRNA in the LPS group was significantly decreased by treatment of 100 mg/kg of GPS (*p* < 0.05). Also, BDNF mRNA levels in the LPS group were decreased significantly compared with those in the SAL group (*p* < 0.05). The decreased levels of BDNF mRNA in the LPS group were significantly increased by administration of 100 mg/kg of GPS (*p* < 0.05). These results indicate that levels of iNOS, TLR4, and BDNF mRNA in the hippocampus of rats given 100 mg/kg of GPS were similar to those of rats given 10 mg/kg IBU.

## 4. Discussion

Our results clearly indicated that GPS treatment significantly improved spatial memory and learning disorders treated by LPS infusions, as evidenced by rat behavior in the MWM test and ORT. GPS also inhibited the increase of IL-1*β* and IL-6 in the hippocampus. In addition, administration of GPS produced increased BDNF mRNA level and decreased iNOS and TLR4 in the hippocampus associated with LPS-treated memory disorder in experimental rats. Therefore, GPS can dose-dependently mitigate LPS-induced cognitive dysfunction via appropriate modulation of NF-*κ*B/iNOS/TLR4/BDNF. Memory impairments induced by the treatments of LPS were analyzed to develop a neuroinflammation rat models [13]. Accordingly, because the production of proinflammatory substances was inhibited in the present study, GPS are effective anti-inflammatory agents and potentially useful neuroprotectants. We investigated the dose-dependent effects of GPS and found that 100 mg/kg of GPS was the most adequate does for preventing LPS-treated negative effects in the MWM test and ORT. In this study, the optimal dose was previously reported [26].

LPS-induced memory dysfunction has been established as a research approach for clarifying the mechanisms underlying the pathophysiology of several neurological disorders such as AD with concurrent and inappropriate behavioral alterations [38]. Neuroinflammation, which may occur after traumatic brain injury, is associated with memory impairments [39]. Accumulating research indicates that persistent inflammation may increase cytokine release, alter protein expression, and, ultimately, disrupt memory networks causing dementia [40, 41]. Therefore, suppression of inflammation may improve cognitive function [39]. Consistent with previous findings [42], those results showed that infiltration of LPS into the lateral ventricle generated significant raises in the inflammatory factors in the rat brains and caused extreme cognitive memory deficiencies.

In the present, administration of GPS after LPS injection markedly recovered body weight, indicating that GPS deceased the physiological changes induced by LPS-induced neuroinflammation [43].

To determine the effects of GPS on two different types of memory, spatial learning and memory and recognition, the behavior tests were used such as ORT and MWM test, respectively. Our results indicated that cognitive memory was impaired under LSP-induced conditions, as observed previously [35]. This impairment was significantly more noticeable after LPS exposure, as evidenced by the significant increase in the exploration time for familiar objects, decrease in the exploration time for novel object, and decrease in the discrimination index. This suggests an intense degeneration in the brain following exposure to memory impairing events, contributing to decreases in episodic memory and recognition ability [35]. In the present study, LPS injection significantly decreased the sniffing time for novel object and reduced the discrimination index. The administration of GPS significantly increased the sniffing time for novel object and improved the impaired recognition memory following LPS injection. The MWM is a hippocampus-dependent memory test, used specifically to demonstrate cognitive impairment and to investigate constant spatial learning capabilities and reference memory in rodents [35]. During the trial sessions of the MWM test, the LPS-infused rats exhibited significantly longer escape latencies to reach the platform and showed deficits in spatial learning. In the LPS-infused rats that received GPS, the rats exhibited faster learning and shorter escape latencies than the nontreated group. Furthermore, the LPS-infused rats without GPS administration performed poorly in successive testing in probe trials 24 h after acquisition, compared with controls, indicating impaired memory recall and retrieval. GPS improved these behavioral abnormalities and restored spatial learning and memory in LPS-induced rats. Long-term treatment with the ibuprofen showed similar effects [35]. Thus, the results in the present study confirm our hypothesis that GPS ameliorates spatial memory impairment induced by LPS injection.

Significant individual differences in locomotor activity and total number of rearing were not observed between the groups, suggesting GPS did not affect sensorimotor performance, motor impairment, or psychomotor function in the OFT. In addition, memory impairment observed in the LPS-infused rats was probably not attributable to differences in their locomotion activities. Consequently, the alterations in behavioral performance in the MWM task were likely to be due to improved memory, not differences in active responses or psychomotor function.

In addition, our studies indicated that LPS infiltration significantly developed TNF-*α*, IL-6, and IL-1*β* expression levels in the hippocampus, ultimately leading to a chronic neuroinflammatory response in the brain. Neuronal damage in the hippocampus generally results in reduced learning and memory ability and is implied in the consolidation of declarative memory in animals and humans [33]. GPS continuously decreased LPS-induced IL-1*β* and IL-6 levels, which finally resulted in improvement from the persistent brain dysfunction and chronic inflammation [40].

Many experimental verification has indicated that the COX-2 is a key element that modulates the proinflammatory mediators including various prostaglandins [44]. The synthesis of prostaglandin E2 (PGE2) and increase of COX-2 and, one of its products, increase in the hippocampus of AD patients [45] may be associated with the pathogenesis of the cognitive impairment and degenerative changes [46]. Our studies indicate that the inflammatory reactions to LPS infiltration significantly stimulated COX-2 mRNA and protein level in the hippocampus by modulating the NF-*κ*B pathway [47]. In several studies, LPS was shown to activate NF-*κ*B, which primarily regulates the expression of inflammatory proteins [48]. In the present study, GPS particularly changed LPS-stimulated behavioral alterations and memory disturbances by inhibiting COX-2 levels, although with nominal statistical significance.

Furthermore, expression of inflammatory genes such as iNOS and COX and activation of NF-*κ*B is inhibited by GPS treatment. Therefore, we hypothesized that GPS may prevent deleterious effects of LPS on memory impairment. In addition, these results may help to explain why GPS may be associated with intracellular NF-*κ*B, which is a major transcription factor that regulates genes responsible for adaptive immune responses [49]. We found the levels of multiple neuroinflammation markers induced by LPS via NF-*κ*B activation, including the LPS receptor TLR4, proinflammatory cytokines, and iNOS implicated in the activation of NF-*κ*B and the production of inflammation mediator, were reduced in GPS-treated rats compared with the controls [50]. TLR4 pathway prevents neurogenesis, which may contribute to the impairment of cognitive functions caused by neuroinflammation [51]. In this study, long-term treatment of LPS activated the NF-*κ*B inflammatory pathway via TLR4 and, thus, triggered the expression of IL-6 and IL-1*β* involved in the hippocampus. Our results demonstrated that GPS could mitigate the induction of proinflammatory mediators including IL-6 and IL-1*β* and iNOS expression caused by LPS. In addition, GPS attenuated LPS-induced inflammatory damage by regulating TLR4/NF-*κ*B responsible for its neuroprotective effects.

We also investigated the BDNF levels, an important factor associated with cognition and memory [39]. Several studies showed that LPS injection reduced hippocampal mRNA levels of BDNF, a neurotrophin that plays a key role in memory and learning processes [52]. BDNF is an important target of NF-*κ*B involved in NMDA receptor-mediated cell survival signaling [38]. Because BDNF plays critical roles in cognitive function and hippocampal synaptic plasticity, maintenance of BDNF signaling is likely to contribute to the beneficial effects of GPS on cognitive function in LPS-injected rats. In the present study, GPS treatment significantly reversed LPS-induced decreases in BDNF mRNA expression, indicating that the beneficial effects of GPS were mediated by increased BDNF mRNA expression, which is potentially associated with enhanced neuronal function and performance in memory tasks.

Interestingly, the LPS + GPS100 group showed no significant recovery against the LPS-induced increase in TNF-*α* and COX-2 levels in the hippocampus, although the same dose of GPS produced statistically significant results in the activation of IL-1*β*, IL-6, and NF-*κ*B levels. The reason that the high dose of GPS did not induce TNF-*α* and COX-2 levels in the LPS-induced neuroinflammation should be investigated further.

## 5. Conclusion

In summary, we demonstrated that GPS significantly improved learning ability and memory in rats with LPS-induced brain dysfunction, as verified by the management on the ORT and MWM test. GPS also inhibited LPS-induced proinflammatory mediators such as IL-1*β* and IL-6 in the hippocampus via modulation of NF-*κ*B/iNOS/TLR4. Based on our results, inflammation may cause cognitive dysfunction by decreasing BDNF gene expression. Collectively, our results indicate that reducing neuroinflammation may be crucial for prevention of dementia and that GPS may provide a useful therapeutic intervention.

## Data Availability

The data used to support the findings of this study are available from the corresponding author upon request.
